# Increased very low frequency pulsations and decreased cardiorespiratory pulsations suggest altered brain clearance in narcolepsy

**DOI:** 10.1038/s43856-022-00187-4

**Published:** 2022-09-30

**Authors:** Matti Järvelä, Janne Kananen, Vesa Korhonen, Niko Huotari, Hanna Ansakorpi, Vesa Kiviniemi

**Affiliations:** 1 Department of Diagnostic Radiology, Medical Research Center (MRC), Oulu University Hospital, Oulu, Finland; 2 Research unit of Medical Imaging, Physics and Technology, the Faculty of Medicine, University of Oulu, Oulu, Finland; 3 Research Unit of Neuroscience, Neurology, University of Oulu, Oulu, Finland; 4 Department of Neurology, Oulu University Hospital, Oulu, Finland

**Keywords:** Sleep disorders, Diseases of the nervous system

## Abstract

**Background:**

Narcolepsy is a chronic neurological disease characterized by daytime sleep attacks, cataplexy, and fragmented sleep. The disease is hypothesized to arise from destruction or dysfunction of hypothalamic hypocretin-producing cells that innervate wake-promoting systems including the ascending arousal network (AAN), which regulates arousal via release of neurotransmitters like noradrenalin. Brain pulsations are thought to drive intracranial cerebrospinal fluid flow linked to brain metabolite transfer that sustains homeostasis. This flow increases in sleep and is suppressed by noradrenalin in the awake state. Here we tested the hypothesis that narcolepsy is associated with altered brain pulsations, and if these pulsations can differentiate narcolepsy type 1 from healthy controls.

**Methods:**

In this case-control study, 23 patients with narcolepsy type 1 (NT1) were imaged with ultrafast fMRI (MREG) along with 23 age- and sex-matched healthy controls (HC). The physiological brain pulsations were quantified as the frequency-wise signal variance. Clinical relevance of the pulsations was investigated with correlation and receiving operating characteristic analysis.

**Results:**

We find that variance and fractional variance in the very low frequency (MREG_vlf_) band are greater in NT1 compared to HC, while cardiac (MREG_card_) and respiratory band variances are lower. Interestingly, these pulsations differences are prominent in the AAN region. We further find that fractional variance in MREG_vlf_ shows promise as an effective bi-classification metric (AUC = 81.4%/78.5%), and that disease severity measured with narcolepsy severity score correlates with MREG_card_ variance (R = −0.48, p = 0.0249).

**Conclusions:**

We suggest that our novel results reflect impaired CSF dynamics that may be linked to altered glymphatic circulation in narcolepsy type 1.

## Plain language summary

The flow of fluid surrounding and inside the human brain is thought to be caused by the movement of brain vessels, breathing and heart rate. These so called brain pulsations are linked to clearing waste from the brain. This process is increased during sleep and suppressed while we are awake. Narcolepsy is a neurological disease where the brain areas regulating being awake and asleep are affected. The diagnosis requires time-consuming hospital tests and is often delayed which has a prolonged negative impact on the patients. Here, we use brain imaging to investigate whether brain pulsations are altered in patients with narcolepsy, and if they can be utilized to differentiate patients with narcolepsy from healthy individuals. We find that narcolepsy affects all brain pulsations, and these findings show promise as an additional diagnostic tool that could help detect the disease earlier.

## Abstract (web summary)

Järvelä et al. investigate if narcolepsy is associated with altered brain pulsations using ultrafast fMRI. They find differences in the brain pulsations between narcolepsy type 1 patients and healthy controls that may link to altered brain clearance in narcolepsy, have diagnostic potential and correlate with the severity of narcolepsy.

## Introduction

Narcolepsy is a debilitating, chronic neurological disease characterized by severe daytime sleepiness, sleep attacks, cataplexy, and fragmented nocturnal sleep^1–3^. The diagnosis of narcolepsy relies on symptoms common to other diseases and tests requiring hospital/sleep center resources including mean sleep latency test, polysomnography, and invasive cerebrospinal fluid (CSF) sampling for analysis of the hypocretin level. Up to 60% of patients with narcolepsy are initially misdiagnosed with, for example, other hypersomnias or depression, and, conversely, up to 46% of initial narcolepsy diagnoses are later found to be misdiagnoses^4,5^. Thus, a correct narcolepsy diagnosis is often delayed for years, which can lead to inappropriate treatment, thus compounding the disease burden in patients with narcolepsy or conditions mistaken for narcolepsy^6,7^.

Narcolepsy type 1 is hypothesized to arise from specific degeneration or dysfunction of the hypocretin-producing cells in the hypothalamus due to an autoimmune reaction that leads to decreased or absent hypocretin signaling in the ascending arousal network (AAN), hypothalamic tuberomammillary nucleus, and throughout the neocortex^2,8^. Hypocretin 1 and 2 (also known as orexin A and B) are 33 and 28 residue peptide neurotransmitters, which have a direct excitatory effect on cortical and brainstem neurons. The AAN of the brainstem comprises neurons of the midbrain reticular formation (MRF), locus coeruleus (LC: noradrenergic), ventral tegmental area and periaqueductal gray matter (VTA, PAG: dopaminergic), and the raphé nuclei (DR: serotonergic), all of which give rise to ascending projections. These AAN innervations participate in the control of autonomic functions and arousal/sleep via their respective neurotransmitters in dense innervations to the hypothalamus, thalamus, basal ganglia, and neocortex^9^. Earlier imaging studies in narcolepsy have shown structural alterations in cortical and subcortical brain regions including the limbic system and brainstem along its nuclei e.g., LC and neocortex^10,11^. Research conducted with task-fMRI have found altered activation of the amygdala, a part of the limbic system, in tasks with humor/reward paradigms along with increased deactivation of the default mode network (DMN) under cognitive burden in narcolepsy^12–14^. Furthermore, a study by Drissi et al. suggests instability of the DMN even in the resting condition^15^. In line with these, in our earlier study utilizing dynamic lag analysis of fast fMRI data at rest, we found the signal propagation as delayed and monotonic, especially between the DMN and other major networks in narcolepsy type 1^16^. Taken together, it is postulated that the degeneration/dysfunction of hypocretin neurons in narcolepsy type 1 manifests in impaired functioning of the AAN, resulting in arousal/sleep disturbances and imbalance in autonomic control of physiology^3,17,18^.

The convection of CSF is driven by physiological brain pulsations, especially cardiorespiratory-related pulsations and vasomotor waves^19^. In a current model of brain fluid homeostasis, CSF water enters the brain parenchyma from arterial perivascular spaces driven by cardiovascular pulsations and exits the brain via paravenous spaces leading to peripheral lymphatic outlets^20,21^. Furthermore, respiration-evoked pressure changes in the thoracic cavity promote venous blood outflow from the brain and reciprocal movement of the CSF from the spinal canal towards the brain, thus facilitating CSF dynamics^22,23^. The CSF flow through the brain parenchyma is an essential aspect of physiology, as the passage of metabolites and immune cells, intracranial fluid dynamics, and brain homeostasis all depend on interstitial CSF flow, which has recently been termed the glymphatic system^24^.

The glymphatic system is most active during sleep and is suppressed by noradrenaline transmission during wakefulness^25^. Moreover, hypertension stiffens arterial walls, thus reducing perivascular pumping and impeding the flow of CSF in perivascular spaces and glymphatic clearance^26^. Furthermore, the reduced noradrenergic tonus prevailing during normal sleep facilitates CSF flow increases in brain interstitium and paravascular spaces^25,27,28^. These findings emphasize the importance of hypocretin, AAN brainstem nuclei, and nocturnal sleep for maintaining brain CSF homeostasis. In the present context, narcolepsy type 1 is characterized by daytime sleep attacks, fragmented nocturnal sleep architecture, a high risk of hypertension, and hypothesized inconsistent noradrenaline transmission in the AAN^1,3,29^. Despite these associations, no previous research has considered whether narcolepsy type 1 impacts brain pulsations and related CSF dynamics.

Only recently it has become possible to non-invasively measure physiological brain pulsations driving CSF and blood flow in the human brain using analysis of fast functional magnetic resonance (fMRI) signal dynamics. Such techniques have revealed marked impairments of the pulsations in patients with Alzheimer’s disease and in epilepsy^30–33^. From a clinical perspective, studies have shown that fMRI signal variability is associated with cognitive performance, increases in dementia, and declines with normal ageing^32,34–36^.

In the present study, we undertake the first investigation of fast fMRI signal variability in narcolepsy type 1 and discuss its relation to CSF dynamics. We use an ultrafast fMRI sequence (MREG: magnetic resonance encephalography) to investigate whether vasomotion-related very low frequency (MREG_vlf_) or individual cardiac (MREG_card_) and respiratory (MREG_resp_) frequency-related brain pulsations are disrupted in narcolepsy type 1. This concept is enabled by the 10 Hz temporal resolution of the modern MREG procedure, which provides temporal signals that are free of aliasing and slice time inaccuracies, thus enabling accurate individual separation of physiological signal instances^37–39^. We further extend our analysis to examine the bi-classification accuracy of these pulsations according to receiving operating characteristic (ROC) and test the clinical association by correlation analysis between brain pulsations and disease severity measured with narcolepsy severity score (NSS)^40,41^. Our a priori hypotheses are that (1) brain pulsations driving CSF flow are disrupted in narcolepsy type 1, (2) brain pulsation measurements can be used to differentiate by non-invasive examination of patients with narcolepsy type 1 and healthy controls, and (3) the extent of pulsation changes in narcolepsy type 1 are associated with disease severity.

Here we find that all three hypotheses are met with confirmatory findings. The very low-frequency pulsations are increased while cardiorespiratory-related pulsations are decreased in narcolepsy type 1. Cardiac-related pulsations in the AAN region negatively correlate with increasing disease severity while very low-frequency pulsations show promise as a bi-classificator. These results imply the clinical relevance of our findings. Taken together, we suggest that our results reflect impaired CSF flow in the brain that may be linked to altered glymphatic function in narcolepsy type 1.

## Methods

### Participants

A registry run from the Oulu University Hospital’s electronic patient records for patients diagnosed with all-type narcolepsy was conducted, resulting in 66 matching diagnoses. All the diagnoses were reassessed with the International Classification of Sleep Disorders (ICSD) 3rd edition^42^ to exclude outdated diagnoses and to confirm narcolepsy type 1 diagnoses according to the latest diagnostic criteria. Twenty-three narcolepsy type 1 patients were recruited for the study. Inclusion criteria were (1) confirmed narcolepsy type 1 diagnosis and (2) clear-cut cataplexy (short, usually bilateral loss of muscle tone without loss of consciousness commonly triggered by emotional stimuli). Other brain-related confounding conditions were excluded by a screening of clinical history and examination of structural MRI brain images. All data were collected between 3/2018 and 9/2020.

Data from one patient was excluded due to corrupted MREG data resulting from the failure of the off-resonance correction during imaging. The blood pressure data from one patient and two controls were missing. The final population consisted of 22 patients with narcolepsy type 1 (NT1 group, 12 females, of mean age of 28.1 ± 8.9 standard deviation (SD)). Four of the NT1 patients were unmedicated and 18 were medically treated for daytime sleepiness and cataplexy (Table 1). All subjects in the NT1 group filled out a disease severity form (NSS: narcolepsy severity scale^40,41^), which ranges from 0 to 57 points. Our patients’ NSS scores ranged from 8 to 45 (mean 26.3 ± 10.5 SD). Twenty-two healthy individuals age- (±3 years) and sex-matched controls without continuous medication were recruited by advertisement from the general population as a control group (HC group, 12 females, of mean age 28.2 ± 8.9 SD). Written informed consent was obtained from all participants. This study was approved by the Ethical Committee of Medical Research in the Northern Ostrobothnia District of Finland and was conducted in accordance with the declaration of Helsinki and GDPR regulations.

**Table 1:** Disease severity by narcolepsy severity score and medication information.

Patients with narcolepsy type 1		
ID	NSS	Medication
1	36	Mo, SSRI
2	25	Mo, Me
3	18	-
4	18	Mo
5	11	Mo
6	13	S
7	45	Me
8	31	S, SNRI
9	28	Me, S
10	31	-
11	33	-
12	22	Mo
13	37	Mo, Me
14	8	Me
15	33	Me, S
16	26	Mo, SNRI
17	20	Mo
18	45	Mo, S, SNRI
19	14	Me
20	19^¤^	Me
21	32	-
22	39	Mo
23	22	Me, S

*ID* subject, *NSS* narcolepsy severity scale score, *Mo* modafinil, *Me* methylphenidate, *S* sodium oxybate, *SSRI* selective serotonin reuptake inhibitor, *SNRI* serotonin–norepinephrine reuptake inhibitor, ^¤^ excluded for corrupted magnetic resonance encephalography data.

### Data acquisition

NT1 and HC participants were scanned with the fast fMRI sequence MREG using a Siemens Magnetom Skyra 3 T MRI scanner (Siemens Healthineers, Germany) with a 32-channel head coil. MREG is a single-shot three-dimensional (3D) sequence that uses a spherical stack of spirals and undersamples the 3D k-space trajectory^37,43,44^. The following parameters were used for the 3D whole brain MREG sequence: repetition time (TR) = 100 msec, echo time (TE) = 36 msec, flip angle (FA) = 25°, field of view (FOV) = 192 mm, voxel size = 3 × 3 × 3 mm^3^. MREG data were reconstructed by L2‐Tikhonov regularization with lambda = 0.1, with the latter regularization parameter determined by the L‐curve method^45^, giving an effective isotropic spatial resolution of 4.5 mm. MREG includes a dynamic off‐resonance in the k‐space method, which corrects for respiration-induced dynamic field‐map changes in fMRI using the 3D single-shot technique^46^. For registration purposes, the participants also had a T1-weighted Magnetization Prepared Rapid Acquisition with Gradient Echo (MPRAGE) scan with parameters as follows: TR = 1900 msec, TE = 2.49 msec, inversion time (TI) = 900 msec, FA 9°, FOV = 240, and slice thickness 0.9 mm). Simultaneously with MREG scanning, end-tidal CO_2_ monitoring and photoplethysmogram were recorded to measure each subject’s respiration and heart rate, respectively.

The subjects were instructed to lie still and awake with eyes open and fixating on a cross on the screen for the entire 10-min resting-state scanning. To minimize head motion and to reduce the effects of scanner noise, soft pads were fitted over the study subjects’ ears, along with earplugs.

### Preprocessing

A preprocessing pipeline from Järvelä et al.^16^ with small changes was implemented here: MREG data were preprocessed with Oxford Centre for Functional MRI of the Brain (FMRIB) software library (FSL, version 5.09) pipeline^47^. The data were high-pass filtered with a cut-off frequency of 0.008 Hz (125 s). To minimize T1-relaxation effects, 180 time points were ignored from the beginning of the data, resulting in 5822 whole brain volumes. Motion correction was carried out using FSL MCFLIRT^47^, and all data and motion parameters were visually inspected for spurious signal fluctuations. The motion was further controlled by the exclusion of any motion exceeding the voxel size (no subject had mean relative motion over 0.07 mm or mean absolute motion over 0.6 mm) and by calculating each subject’s mean frame-wise displacement. Brain extraction for 3D MPRAGE volumes was performed with the FSL Brain Extraction Tool using neck and bias-field correction and the following parameters: fractional intensity = 0.20–0.22 and threshold gradient = 0.05–0.25. To obtain optimal quality, the extracted brain images were visually inspected. Images were spatially smoothed with a 5 mm full width and half maximum (FWHM) Gaussian kernel using *fslmaths*. MREG images were aligned to the 3D anatomical images (full‐search, 12 degrees of freedom (DOF)) and to the Montreal Neurological Institute (MNI152) 4 mm standard space (full‐search, 12 DOF) as a preprocessing step in the FSL multivariate exploratory linear optimized decomposition into independent components tool.

### Brain pulsation range estimation

**Fig. 1:**
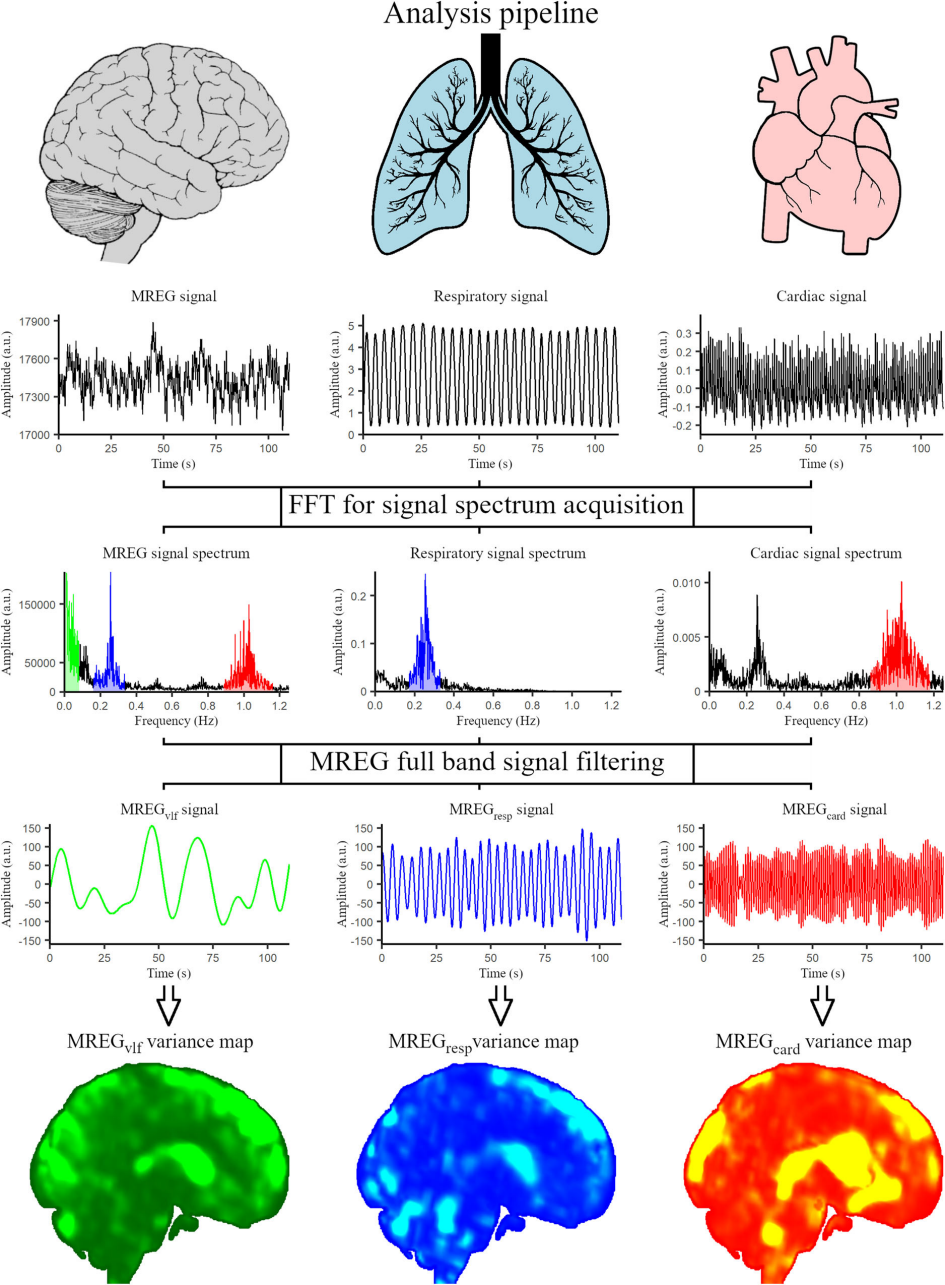
Analysis pipeline. Temporal magnetic resonance encephalography (MREG), respiratory (end-tidal CO_2_), and cardiac (photoplethysmogram) signals are transformed into frequency spectra with fast Fourier transformation (FFT). Individual minimum, maximum, and peak values for respiration (blue) and cardiac (red) frequencies are obtained and the cardiorespiratory ranges are calculated. MREG full band signal is filtered to these physiological ranges and to very low frequency (green). Voxel-wise variance maps are calculated for each MREG frequency band. MREG_vlf_ very low-frequency filtered MREG, MREG_resp_ respiratory frequency filtered MREG, MREG_card_ cardiac frequency filtered MREG.

Individual respiratory and cardiac frequencies were extracted from end-tidal CO_2_ and photoplethysmogram data with the MATLAB version R2019b fast Fourier transformation (FFT) *fft* function. From the signal frequency spectrum, respiratory and cardiac minimum, maximum, and peak values were obtained (Fig. 1). Minimum values were subtracted from the maximum values to calculate individual cardiorespiratory frequency ranges.

In cases with corrupted physiological data (5 end-tidal CO_2_ and 11 photoplethysmogram data recordings), the MREG data were used to estimate physiological frequency ranges. First, Analysis of Functional NeuroImages’ (AFNI, version 18.0.05)^48^ *3dPeriodogram* was used to transform the preprocessed four-dimensional MREG data into a voxel-wise FFT spectrum. For respiratory and cardiac frequency estimation, the fourth ventricle and anterior/middle cerebral arteries, respectively, were chosen as reference points, as the investigated physiological events in the MREG FFT spectrum were visually most pronounced in these regions. The corresponding minimum, maximum, and peak values for cardiorespiratory frequencies were then estimated. To test the precision of the estimation of the MREG FFT spectrum, minimum, maximum, and peak frequencies were extracted from 41 available end-tidal CO_2_ and 35 photoplethysmogram recordings and correlated with their MREG FFT spectrum counterparts using the R software *ggscatter* function (Supplementary Fig. 1). The MREG FFT spectrum estimations of cardiorespiratory frequencies were found to be accurate when compared with the gold standard estimators, and were thus used in further analyzes in those cases where the peripheral physiological data were corrupted or missing. While estimating respiratory ranges across groups, the minimum value of respiration was found to be lower than 0.1 Hz in two NT1 subjects. To avoid any confounding effects of respiration on very low-frequency variance calculation, the upper limit of the very low-frequency band was set to 0.08 Hz, paralleling previous research^49–51^.

### Variance analysis

The general workflow is described in Fig. 1. To investigate individual physiological pulsation spectrum, AFNI *3dTproject* was used to bandpass-filter all the preprocessed data sets to very low frequency (0.01–0.08 Hz), and individual respiratory and cardiac frequencies. Signal variance is a measure of variability and probability distribution, defined as the expectation of the squared deviation of a random variable from its mean:

1 $${{{{{{\mathrm{Variance}}}}}}}\,=\,\frac{{\Sigma ({\chi }_{i}-\mu )}^{2}}{N-1}$$

where χ_i_ is the variable (signal amplitude value at a given time), µ is the signal mean, and N is the number of time points.

Fractional variance (Var_frac_) is a measure used, for example, in principal component analysis (indicating the percentage of variance explained by a component)^52^, which is defined as the variance of a random variable divided by the total variance of the measurement:

2 $${{{{{{{\mathrm{Var}}}}}}}}_{{{{{{{\mathrm{fraq}}}}}}}}=\,\frac{{{{{{{{\mathrm{Var}}}}}}}}_{i}}{{{{{{{{\mathrm{Var}}}}}}}}_{t}}$$

where Var_frac_ is fractional variance, Var_i_ is partial signal variance, and Var_t_ is total signal variance. The variance was chosen as the measure of signal variability rather than the standard deviation and coefficient of variation, as used in the previous research^31,32,53^ as these latter measures cannot be used to calculate proportional variability of different frequency bands.

Brain voxel-wise variance was calculated with FSL *fslmaths* for full band (0.008–5 Hz) time series and all physiological pulsation frequencies. Then, all voxel-wise variance data were registered to the MNI152 3 mm standard space with FSL *flirt*^54^ and masked with an MNI152 3 mm standard brain binary mask to remove any residual MREG voxels outside the co-registered standard space. The Var_frac_ values were obtained by dividing the very low frequency, respiratory, and cardiac frequency variances with the full band variance and then applying the same processing steps described above to acquire brain vowel-wise maps in standard space. Finally, variance and Var_frac_ maps were compared between HC and NT1 groups with 10,000 iterations of FSL *randomize* to extract the corrected *p* value t-statistic maps (*p* < 0.05)^55^. The results were then displayed on top of the MRIcroGL MNI152 standard brain.

### Correlation and receiving operating characteristic analyses

AAN in 1 mm MNI152 standard space mapped by Edlow et al.^8^ was used as a hypothesis-driven ROI. Variance results were first registered to 1 mm MNI152 standard space. A whole AAN ROI spanning 2042 voxels/mm^3^ was generated by creating a binary mask of individual AAN nuclei (for example, LC, MRF, VTA as defined by Edlow et al.) with *fslmaths*, and then applied to the variance maps. Individual nuclei remain small but at the group level, MREG has been shown to be capable of delineating minimal respiratory centers even in the relatively pulsatile brainstem^56^. The correlation between disease severity (NSS score) and mean variances from the AAN ROI were calculated with the RStudio version 1.3.1093 software *ggplot2* library commands. ROC curves were plotted with the R software *pROC* library commands, and also applied to individual brainstem areas comprising the AAN. The final editing of the figures was done with GNU Image Manipulation Program version 2.10.30.

### Statistics and reproducibility

All analyses were conducted between the 22 subjects in the NT1 group and the 22 subjects in the HC group except between the blood pressure measurements where the available 21 NT1 and 20 HC were used for the comparisons, and when correlating end-tidal CO_2_ and photoplethysmogram derived cardiorespiratory frequencies to the corresponding MREG data (41 end-tidal CO_2_ vs. MREG pairs and 35 photoplethysmogram vs. MREG pairs). The normality of the data distributions was estimated visually and with Shapiro–Wilk test. Two-tailed Student’s *t*-test and Wilcoxon rank-sum test were used for hypothesis testing between the study groups in cardiorespiratory frequency estimation (minimum, maximum, peak values, and range), motion, blood pressure (significant *p* value < 0.05), and individual nuclei variance results that were further corrected for multiple comparisons with the Benjamini–Hochberg method (significant *p* value < 0.033 or 0.016, please see Supplementary Tables 1, 2). Wilcoxon and Cohen’s d effect sizes were calculated with Rstudio’s *wilcoxon_effsize* and *cohens_d* functions. Pearson correlation coefficient was used to determine association between NSS and variance results (significant *p* value < 0.025). The data describing the study population is available in Supplementary Tables 1, 2. *Randomize* uses conditional Monte Carlo random permutations implementing family-wise error-corrected threshold-free cluster enhancement correction in both directions (HC > NT1, HC < NT1) separately^55^, thus taking into account the multiple comparisons, and was used to test for differences between the study groups in variance and fractional variance maps (significant *p* value <0.05).

### Reporting summary

Further information on research design is available in the Nature Research Reporting Summary linked to this article.

## Results

### Pulsation spectrum analysis

**Fig. 2:**
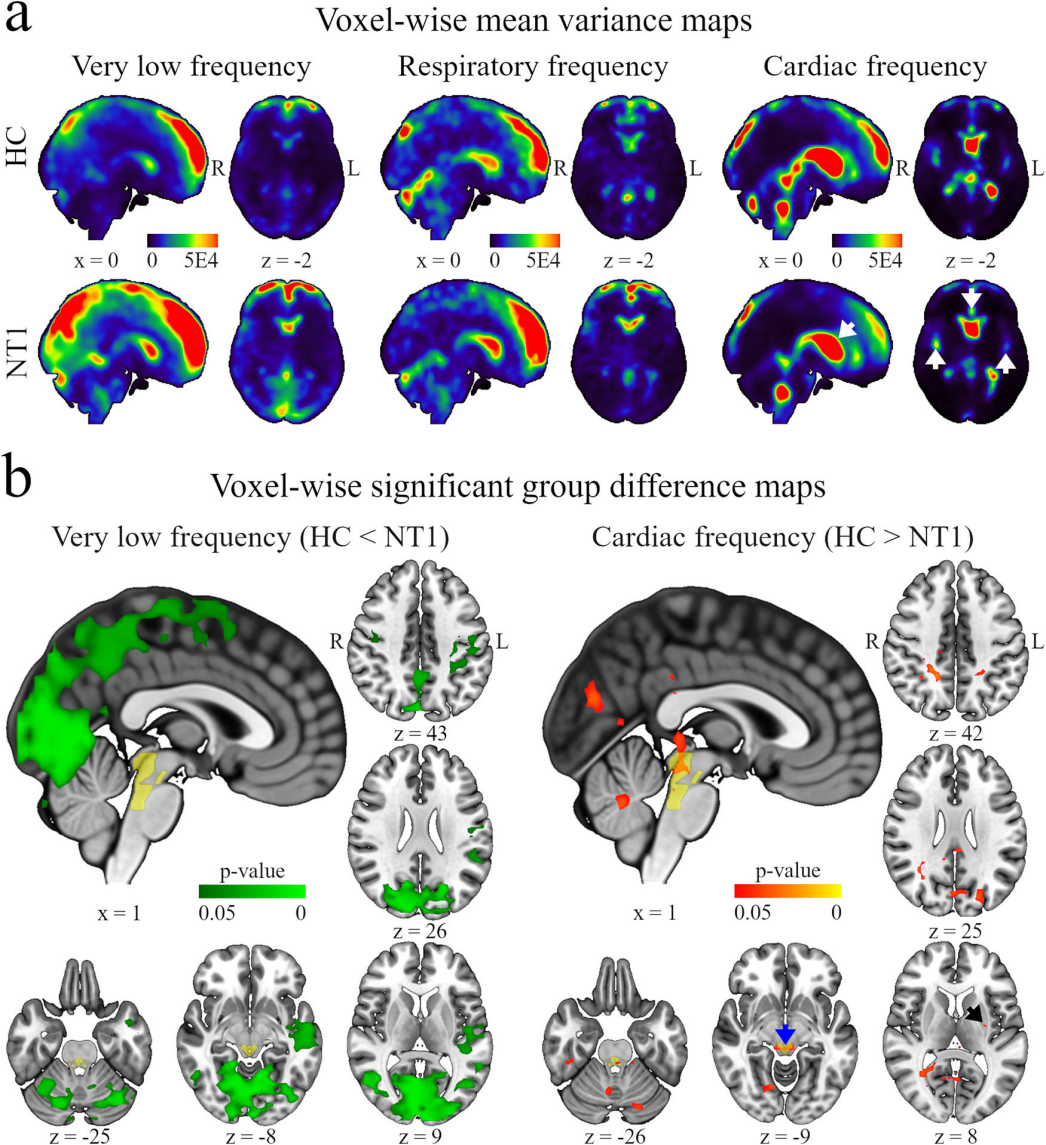
Frequency-wise variance results. **a** Voxel-wise mean-variance maps. Very low frequency- and respiratory-related variances mostly populate anterior and posterior cortical regions, while cardiac-related variance dominates in the ventricular system and large arteries. White arrows = anterior-, middle cerebral arteries, and ventricular system. Color bars represent a sliding scale of mean-variance values across all healthy controls (HC) and patients with narcolepsy type 1 (NT1), respectively. R right, L left, x displayed sagittal plane, z displayed axial plane. **b** Voxel-wise significant group difference maps. Very low-frequency results locate (HC < NT1, green area) mostly in the posterior brain areas, while cardiac-related results (HC > NT1, red area) interestingly overlap with ascending arousal network (AAN). Black arrow =;left basal ganglia, blue arrow = aqueductus sylvii, yellow area = AAN. *P* values < 0.05 are significant, *n* = 22 HC vs. 22 NT1.

**Fig. 3:**
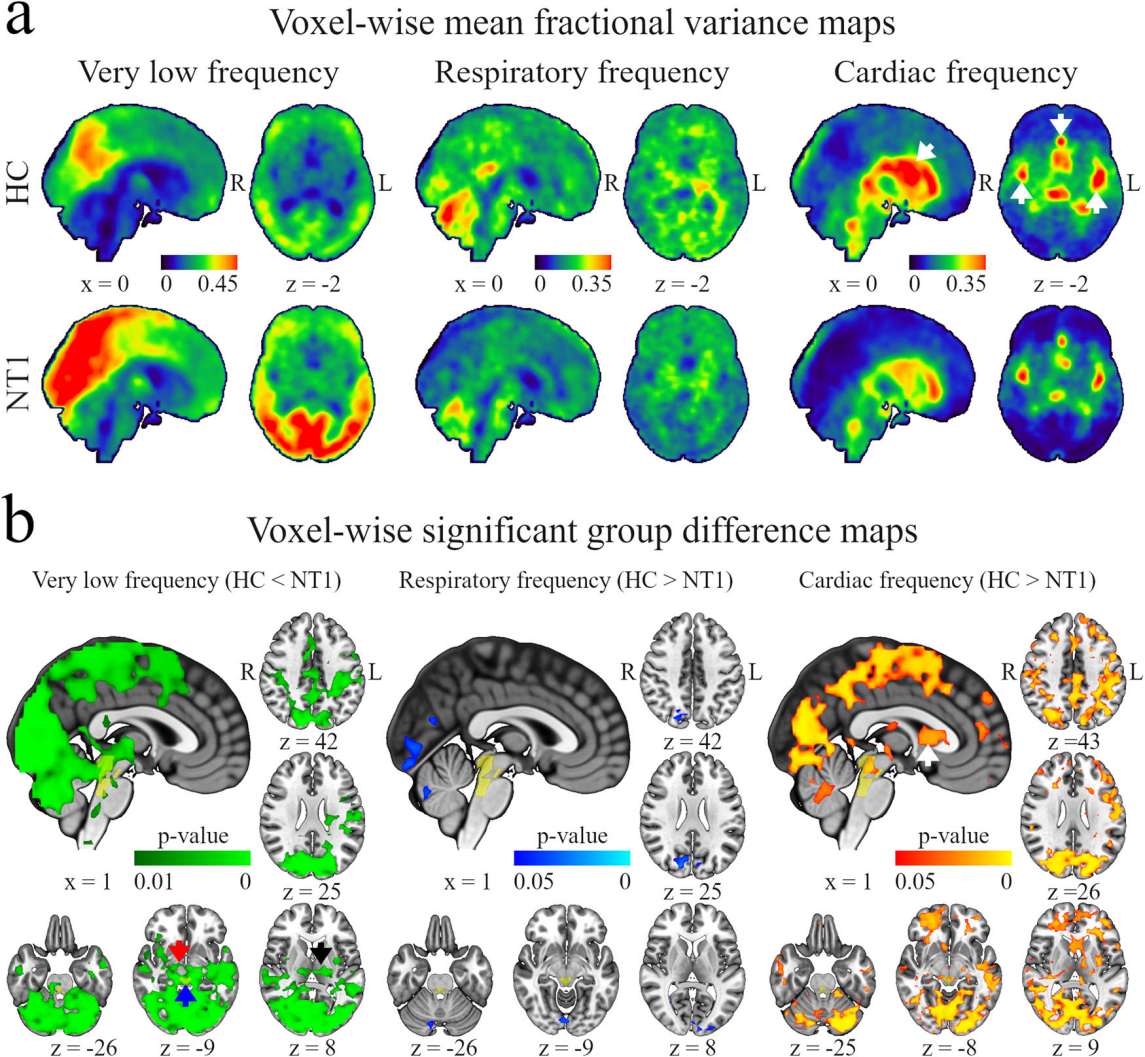
Frequency-wise fractional variance results. **a** Voxel-wise mean fractional variance maps. Very low frequency- and respiratory-related fractional variance are most pronounced in the brain parenchyma, while cardiac-related fractional variance populates the ventricular system and large arteries. White arrows = anterior-, middle cerebral arteries, and ventricular system. Color bars represent a sliding scale of mean fractional variance values across all healthy controls (HC) and patients with narcolepsy type 1 (NT1), respectively. R right, L left, x displayed sagittal plane, z displayed axial plane **b** Voxel-wise significant group difference maps. Fractional variance results in very low frequency (HC < NT1, green area), respiratory-related (HC > NT1, blue area), and cardiac-related (HC > NT1, red area) pulsation frequencies overlap mostly in the posterior brain regions and ascending arousal network (AAN). White arrow = anterior corni of the lateral ventricles, black arrow = basal ganglia, blue arrow = aqueductus sylvii, red arrow = hypothalamus, yellow area = AAN. *P* values < 0.05 are significant, *n* = 22 HC vs. 22 NT1.

To investigate the brain pulsation spectrum, we compared brain voxel-wise variance and Var_frac_ in MREG_vlf_ and individual MREG_resp_ and MREG_card_ between the HC and NT1 groups (Figs. 2 and 3). In both groups, most of the very low frequency and respiratory-related variance was found in anterior and posterior cortical areas, while cardiac-related variance was more pronounced in ventricular CSF spaces and in anterior and middle cerebral arteries (Fig. 2a). In between-group analysis, we found that variance in MREG_vlf_ was higher in the NT1 group compared to HC in a large posterior cortical region covering primary visual, lingual and intracalcarine cortices and, furthermore in the left temporal gyri (total volume of 194 cm^3^, Fig. 2b). Interestingly, variance in MREG_card_ was lower in the NT1 group compared to HC in parts of left putamen and amygdala, along the aqueductus sylvii and in the AAN ROI (total volume of 21 cm^3^), a region engaged in sleep-wake control and excitation of the cortex (Fig. 2b). An overlap with variance results between MREG_vlf_ and MREG_card_ was most pronounced in the left intracalcarine and lingual cortices (total overlap of 5 cm^3^). We found no group differences in MREG_resp_ variance. Our results suggest greater very low frequency and decreased cardiac-related brain pulsations in narcolepsy type 1 compared to healthy individuals.

To explore how strongly different brain pulsations explain the observed total signal variance (i.e., the proportional variability of the pulsations), we calculated brain voxel-wise Var_frac_ maps in different pulsation frequency ranges (Fig. 3). Here, most of the very low-frequency fractional variance in both groups was located in the cerebral cortex with lesser involvement of the ventricular system while most of the respiratory-related fractional variance was spread more centrally yet pervasively in brain tissue, but avoiding most ventricular CSF spaces and prominent arteries. Contrasting with the previous, most of the cardiac-related fractional variance was found in the ventricular system and in the anterior and middle cerebral arteries (Fig. 3a). Mirroring the above group-wise results, we found that MREG_vlf_ Var_frac_ was higher in the NT1 group compared to HC in a large portion of the posterior cortices. However, these results were more robust and widespread (total volume of 855 cm^3^), also encompassing the bilateral thalami, putamen, insular cortices, and, interestingly, AAN and right hypothalamus—both of which are implicated in narcolepsy type 1 pathology (Fig. 3b). Furthermore, MREG_card_ Var_frac_ was lower in the AAN, bilateral thalami, caudate nuclei, left putamen, anterior corni of the lateral ventricles, left temporal and right frontal lobe, primarily visual, lingual, and intracalcarine cortices of the NT1 group (total volume of 428 cm^3^, Fig. 3b), thus including both parenchymal and CSF loci and overlapping the MREG_vlf_ Var_fraq_ results in a volume totaling 315 cm^3^. Var_frac_ in MREG_resp_ was lower in the left lingual and intracalcarine cortices of the patients (total volume of 14 cm^3^, Fig. 3b) completely overlapping with the MREG_vlf_ Var_fraq_ results and overlapping in with the MREG_card_ Var_fraq_ results in a volume totaling 10 cm^3^. Our findings suggest that the defined physiological pulsations differ in distinct proportions in narcolepsy type 1 patients compared to the healthy population.

**Fig. 4:**
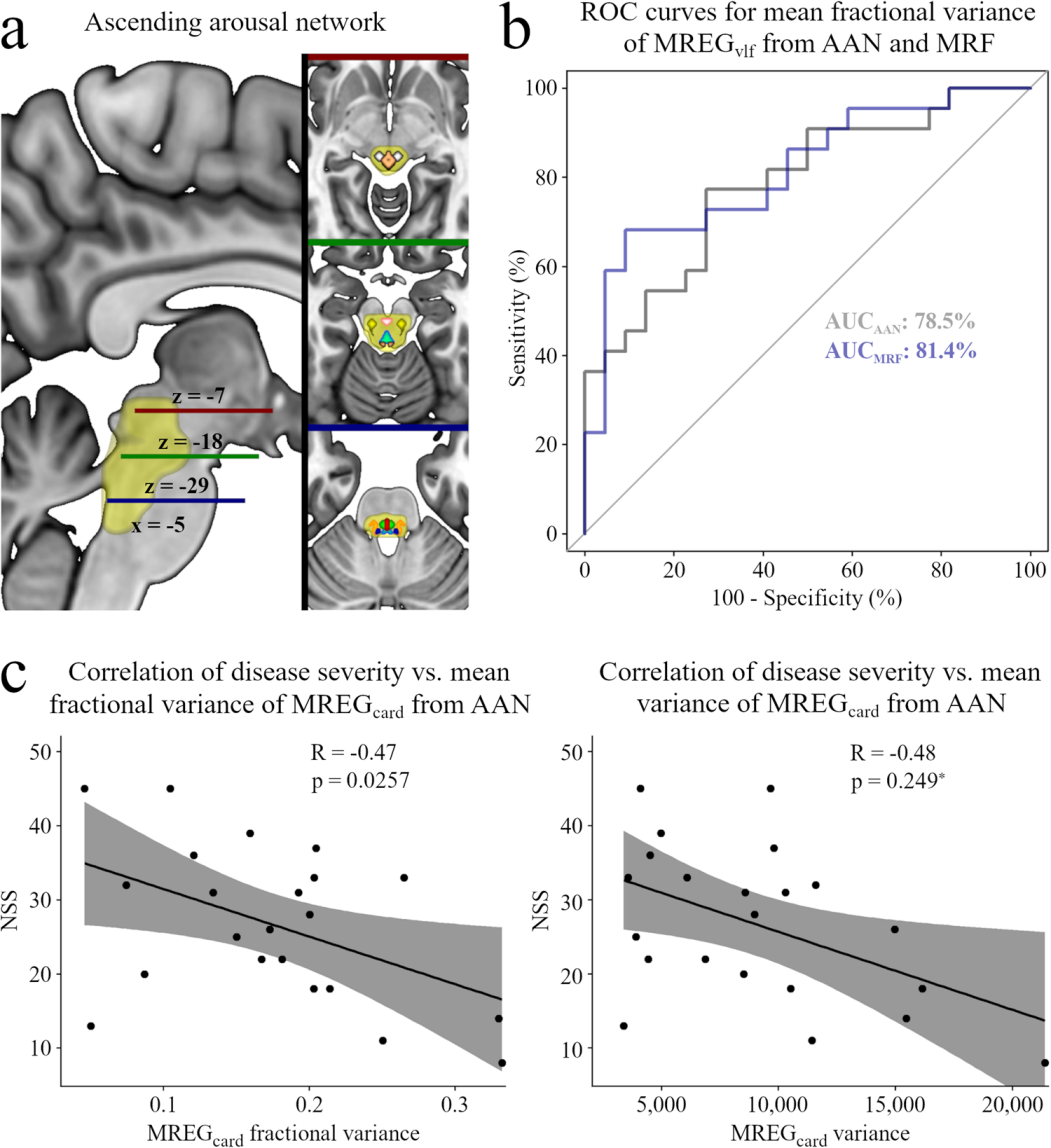
ROC and correlation analysis results. **a** Ascending arousal network (AAN, light yellow). z = −7 (red line): periaqueductal gray (PAG, copper), midbrain reticular formation (MRF, white). z = −18 (green line): ventral tegmental area (VTA, light red), pedunculopontine nucleus (PPN, yellow), dorsal raphé (DR, light blue). z = −29 (blue line): pontis oralis (PO, green), parabrachial complex (PBC, orange), median raphe (MR, red), locus coeruleus (LC, dark blue), x = displayed sagittal plane, z = displayed axial plane. **b** Receiver operating characteristic area under the curve (ROC AUC) for mean fractional variance at a very low frequency of the magnetic resonance encephalography (MREG_vlf_) in the AAN (gray) and MRF (blue) regions shows promise as a bi-classifier. **c** Correlation between disease severity (NSS narcolepsy severity scale) and cardiac frequency filtered MREG data (MREG_card_) variance results indicate the clinical relevance of the method. Black dots denote individual data points and gray area indicates a 95% confidence interval. R Pearson correlation coefficient, * = significant *p* value < 0.025, *n* = 22 vs. 22.

To analyze the clinical relevance of our findings, we investigated ROC curves and calculated correlations between disease severity represented by NSS scores and our variance results from the AAN, which was our hypothesis-driven ROI (Fig. 4a). Analyzes of ROC curves from the AAN and its nuclei revealed that brain pulsation findings in MREG_vlf_ could effectively discriminate narcolepsy 1 patients from a healthy population (AUC = 81.4% in MREG_vlf_ Var_frac_ for MRF and 78.5% for the AAN, Fig. 4b). The mean Var_frac_ in MREG_vlf_ was significantly higher in all AAN nuclei except VTA of the patient group, and Var_frac_ in MREG_card_ was lower in DR, MRF and PAG divisions of the AAN (Supplementary Tables 1 and 2). Furthermore, we observed decreased MREG_card_ variance in the MRF of the patients. Interestingly, unlike in MREG_vlf_ Var_frac_, we found no group differences in MREG_vlf_ variance within the AAN. We further found significant negative correlation and strong trend-wise negative correlation between MREG_card_ mean-variance results in AAN and NSS scoring (mean = 9053 ± 4784 SD; 20 degrees of freedom (df); T = −2.4238; 95% confidence interval (CI) [−0.75, −0.067]; R = −0.48; *p* = 0.0249 for mean-variance, and 0.17 ± 0.078 SD; 20 df; T = −2.4101; 95% CI [−0.75, −0.066]; R = −0.47, *p* = 0.0257 for mean Var_frac_ in MREG_card_, Fig. 4c). The ROC and correlation analyses suggest that our variance results may have clinical applications.

### Individual cardiorespiratory frequencies, motion, and blood pressure

We observed no significant differences between the NT1 and HC groups in respiratory (median = 0.12 [first quartile (Q1): 0.10, third quartile (Q3): 0.14] for the NT1 and 0.12 [Q1: 0.11, Q3: 0.13] for the HC; W = 224.5; 95% CI [−0.018, 0.012]; Wilcoxon rank sum *p* = 0.69) or cardiac frequency ranges (median = 0.24 [Q1: 0.20, Q3: 0.29] for the NT1 and 0.26 [Q1: 0.19, Q3: 0.34] for the HC; W = 217; 95% CI [−0.079, 0.038]; Wilcoxon rank sum *p* = 0.57). Furthermore, there were no significant group differences in individual respiratory minimum (mean = 0.21 ± 0.052 SD for the NT1 and 0.19 ± 0.057 SD for the HC; 42 df; T = 1.1505; 95% CI [−0.014, 0.052]; Student’s *t*-test *p* = 0.26), maximum (mean = 0.33 ± 0.061 SD for the NT1 and 0.31 ± 0.059 SD for the HC; 42 df; T = 0.72697; 95% CI [−0.023, 0.050]; Student’s *t*-test *p* = 0.47) and peak (mean = 0.27 ± 0.063 SD for the NT1 and 0.25 ± 0.063 SD for the HC; 42 df; T = 1.3011, 95% CI [−0.014, 0.063]; Student’s *t*-test *p* = 0.20) values or cardiac minimum (mean = 0.94 ± 0.13 SD for the NT1 and 0.91 ± 0.097 SD for the HC; 42 df; T = 0.87337; 95% CI [−0.040, 0.10]; Student’s *t*-test *p* = 0.39), maximum (mean = 1.2 ± 0.12 SD for the NT1 and 1.2 ± 0.14 SD for the HC; 42 df; T = −0.28269; 95% CI [−0.091, 0.069]; Student’s *t*-test *p* = 0.78) and peak (mean = 1.0 ± 0.13 SD for the NT1 and 1.1 ± 0.10 SD for the HC; 42 df; T = −0.35701; 95% CI [−0.083, 0.058], Student’s *t*-test *p* = 0.72) frequencies. Group differences were absent in the mean absolute movement (median = 0.17 [Q1:0.13, Q3: 0.27] for the NT1 and 0.16 [Q1: 0.12, Q3: 0.22] for the HC; W = 289; 95 CI [−0.021, 0.075]; Wilcoxon rank sum *p* = 0.28), mean relative movement (median = 0.027 [Q1: 0.024, Q3: 0.031] for the NT1 and 0.030 [Q1: 0.024, Q3: 0.035] for the HC; W = 201; 95% CI [−0.0071, 0.0028]; Wilcoxon rank sum *p* = 0.35) and frame-wise displacement (median = 0.031 [Q1: 0.023, Q3: 0.038] for the NT1 and 0.028 [Q1: 0.024, Q3: 0.033] for the HC; W = 275; 95% CI [−0.0038, 0.0081]; Wilcoxon rank sum *p* = 0.45). No significant differences were observed in systolic (mean = 127 ± 15 SD for the NT1 and 131 ± 18 SD for the HC; 39 df; T = −0.66572; 95% CI [−14, 7.1]; Student’s *t*-test *p* = 0.51) and diastolic (mean = 79 ± 9.4 SD for the NT1 and 77 ± 12 SD for the HC; 39 df; T = 0.67313; 95% CI [−4.5, 9.0]; Student’s *t*-test *p* = 0.50) blood pressure or mean arterial pressure (mean = 95 ± 10 SD for the NT1 and 95 ± 13 SD for the HC; 39 df; T = 0.091909; 95% CI [−7.2, 7.9], Student’s *t*-test *p* = 0.93). These results imply that our variance results are not attributable to group differences in cardiorespiratory function, motion, or blood pressure. Additionally, the lack of group-wise difference in these peripheral estimators suggests that our fMRI variance results do not arise from peripheral sources such as respiration and pulse per se, but rather are intrinsic to the brain.

## Discussion

In this study, we used ultrafast fMRI to determine whether the brain pulsation spectrum characterized as signal variability in very low frequency, respiratory, and cardiac frequency domains differ in patients with narcolepsy type 1 versus healthy subjects, and if signal variance could differentiate patients from healthy controls. Supporting our hypotheses, we found that, compared to controls, the NT1 group had (1) higher variance and Var_fraq_ in MREG_vlf_ but the lower variance and Var_fraq_ in MREG_card_ and in MREG_resp_ Var_fraq_. The results in MREG_vlf_ and MREG_card_ encompassed wide posterior brain cortical, CSF, and brainstem regions, including our a priori target region AAN, which is known to be implicated in the pathology of narcolepsy^9^. (2) A ROC AUC analysis of mean Var_frac_ in MREG_vlf_ from MRF and whole AAN ROIs had good properties as a potential diagnostic bi-classifier (AUC = 81.4% for MRF and 78.5% for AAN), and (3) disease severity correlated negatively with mean cardiac-related variance measures (R = −0.48, *p* = 0.0249 for mean-variance and R = −0.47, *p* = 0.0257 for mean Var_frac_ in MREG_card_), indicating a degree of clinical relevance. Taken together, our results imply that parenchymal and ventricular CSF flow may be impaired in narcolepsy type 1, which may be linked to altered metabolite transfer in the disease. We conclude that based on earlier findings and literature, our present findings of altered brain pulsations in narcolepsy type 1 most likely relate primarily to hypocretin deficiency and the consequent lack of hypocretin signaling on the downstream AAN and cortical regions, which in turn underlies possible autonomic alterations and altered sleep-wake patterns characteristic of narcolepsy type 1.

Earlier studies have shown that low-frequency blood oxygenation level-dependent (BOLD) signal fluctuations are related to hemodynamic oscillations that mirror neuronal activity^57^. In contrast, a proportion of the very low-frequency oscillations has been attributed to vascular slow sinusoidal hemodynamic shifts driven by slow vasomotor waves^58–61^. Vasomotion is in turn postulated to drive perivascular CSF flow in the brain parenchyma^19,62^. Studies in mice and men show that physiological intracranial processes connected to intrathoracic functions affect brain CSF dynamics and brain clearance^20,22^. Cardiac cycle-related brain arterial pulsatility, which is moderated by vasomotor activity in arterial smooth muscles, facilitates the bulk flow of CSF from perivascular spaces into the brain interstitium, which later drains to paravenous spaces and ultimately to the peripheral lymphatics^21,26^. Furthermore, respiration induces intrathoracic pressure changes via venous outflow from the brain, which in turn causes inflow of CSF from the spinal canal to the brain and thus contributes to parenchymal (para)vascular fluid transport^23^. Thus, intracranial and parenchymal CSF flux is thought to be driven by very low frequency, cardiac-, and respiratory-related brain pulsations that arise respectively from vasomotion/hemodynamics, cardiac-induced arterial pulsations, and breathing^19^.

Our results for MREG_vlf_ signal variance and Var_frac_ were robust in the posterior cerebral cortices and brainstem (Figs. 2 and 3). Very low-frequency fluctuations of the BOLD signal are related to vasomotor activity, i.e., vasodilation/contraction in arterioles^58–61^. In the awake brain, neuronal activation induces vasodilation, while in sleep the vasomotor waves are induced by systemic blood pressure waves^63–65^. In mice, there is a link between neuronal activation-driven vasodilation with increased paravascular tracer clearance^62,66^. Based on recent findings and our present results, the increased MREG_vlf_ variability we observed in the NT1 group may partly reflect altered vasomotor drive of parenchymal CSF in narcolepsy type 1. This is supported by a recent computational model study concluding that neuronal activation and associated vasodilatation and hyperemia, and the consequent flow through the vascular basement membrane and artery wall, may contribute to brain metabolite clearance^67^. The proposition is further supported by Fultz et al., who found that slow oscillations in neuronal activity, blood oxygenation, and CSF flow are coupled in sleep, leading the authors to postulate a mechanism whereby slow neuronal activity contributes to metabolite clearance^68^. Increased MREG signal variability reflects more extreme shifts around the signal mean, and decreased variance indicates more moderate shifts. In the present context, altered signal variability is thought to propagate to altered flow/pumping of blood and CSF in the brain parenchyma and ventricular system. Of note, Var_frac_ in MREG_vlf_ in the NT1 group was increased in the hypothalamus; the primary site of the hypocretin-producing cells, which in the human brain normally number some 100,000, but are presumably depopulated by a specific autoimmune reaction in narcolepsy type 1, thus causally leading to the disease^2,69^. The pathophysiology underlying our fMRI results likely relates to downstream consequences of a failure of hypocretinergic regulation of ascending AAN projections. Interestingly, we found that the NT1 group had increased MREG_vlf_ Var_frac_ in all AAN areas except VTA compared to the HC group, including the LC and aqueductus sylvii, thus overlapping with our cardiac-related results (Fig. 3). This may reflect a change in the pulsation spectrum towards very low frequencies, to the detriment of cardiac-related pulsation. These opposing changes may indeed be pathologically connected to narcolepsy type 1 given that an increase in vasodilation coupled with very low-frequency signal power in the healthy brain is associated with increased cardiac-related power^70^. Thus, we speculate that, in the context of brain hydrodynamics, decreased and increased pulsation amplitudes when compared to a healthy population may likewise weaken brain fluid mechanics.

Our results indicate decreased variance in cardiac frequency pulsations for NT1 patients throughout the AAN, including also LC and aqueductus sylvii, which is the route for CSF flow between the third to the fourth ventricles (Fig. 3b). Furthermore, the NT1 group had lower Var_frac_ in MREG_card_ in the anterior *corni* of the lateral ventricles. Since the cardiac pulsation is known to drive CSF flow also in the ventricular system^71–73^, these present results may reflect the altered flow of CSF along the aqueduct and through the lateral ventricles in narcolepsy type 1 patients. A recent study showed that interstitial brain volume increases during sleep, while noradrenaline activity decreased this volume during wakefulness, indicating that declining noradrenaline-driven vasomotor tone during sleep is conducive to CSF flow through the brain^25^. In narcolepsy type 1, the presumed loss of hypocretin-producing cells of the hypothalamus deprives the AAN nuclei of an excitatory input, which may lead to postulated inconsistent neurotransmitter release from these nuclei, notably the noradrenergic LC. Interestingly, we found decreased Var_frac_ in MREG_card_ in parts of the cortical gray and white matter of the patient group (Fig. 3b). We speculate that this decreased proportional signal variance is due to a shift towards a lower frequency band (in regions overlapping with the increased MREG_vlf_ variance in the same NT1 group). This may reflect lower intracortical arterial pulsatility, which plausibly propagates to reduced CSF bulk flow from perivascular spaces. Based on previous research on the functional neuroanatomy of the AAN nuclei, we speculate that these variance findings are consistent with perturbed brainstem noradrenergic regulation arising downstream from a primary defect in the hypocretin system.

We found no difference in MREG_resp_ variance between the NT1 and HC groups. However, Var_frac_ in MREG_resp_ was lower in regions encompassing the left lingual gyrus and intracalcarine cortices in the NT1 group (Fig. 3b), implying that the proportion of variance arising from respiratory pulsation was reduced in favor of other frequency bands. As the respiratory-related results co-localized exclusively with increased very low-frequency variance results in the NT1 group, very low-frequency pulsations in these regions may dominate over their respiratory counterparts in narcolepsy type 1. This perturbation may propagate to the altered flow of intraparenchymal CSF, as respiration is considered a major driving force of CSF flow^22^. Considering the different pulsation range variance results, we find that Var_frac_ may be a more robust indicator of pathological pulsations than plain variance, since the findings in Var_frac_ are more pronounced and widespread (Figs. 2 and 3).

Our present cardiac- and very low frequency -related results partly overlap with an earlier task- and resting-state imaging findings in patients with narcolepsy. In two studies, Drissi et al. found that in patients with narcolepsy the dynamic resting activity of the DMN is unstable and that a lower R2 relaxation rate in the brainstem’s rostral reticular formation may indicate lower neuromelanin levels in the LC in narcolepsy when compared to the healthy population^11,15^. Moreover, in narcolepsy increased deactivation of the DMN has been observed under cognitive burden induced by a working memory test suggesting dysregulation of sustained attention by an imbalance in the utilization of cognitive resources^14^. In accordance with the earlier imaging studies, we found that the temporal dynamics of the very low-frequency fMRI signal propagation between the DMN and other resting-state networks are slow/monotonic as characterized by altered distribution of inter-network causal lags thus reflecting both altered activation and deactivation of the DMN at rest in narcolepsy type 1 when compared with healthy controls^16^. These studies offer robust evidence that in narcolepsy, DMN activity is affected. However, the DMN does not exist in a vacuum devoid of the vasomotor element in very low frequencies. This is demonstrated by our present results covering the posterior cingulate cortex/precuneus, i.e., the main components of the posterior DMN, but also widespread brain regions beyond the DMN without clear delineation to any specific resting-state network (Figs. 2 and 3). Thus, in narcolepsy type 1 not only is the DMN activation patterns affected but the brain-wide pulsations including the DMN may be altered. However, as functional connectivity analyses are correlation-based and mostly static, the comparison with present brain pulsation findings may warrant more thorough analyses in the future.

Interestingly, we observed no difference between the NT1 and HC groups in cardiac or respiratory rates per se. Yet, we found that cardiac-related variance correlated with disease severity. We speculate that, while respiration and cardiac pulse are crucial for intracranial CSF flow, a pathology of cerebral pulsation is not evident from direct cardiorespiratory frequency measurements. Rather, we suggest that the pulsation disturbances in the NT1 group are intrinsic to the brain. This position is supported by analogous findings in Alzheimer’s disease and epilepsy^31,32^. Importantly, we found no differences between the NT1 and HC groups in full band MREG variance measures, probably due to a juxtaposition of increased very low frequency and decreased cardiorespiratory variances, which underlines the importance of accurate pulsation characterization with the ultrafast fMRI technique.

Narcolepsy type 1 is diagnosed from clinical phenotype (symptoms), mean sleep latency test accompanied by nocturnal polysomnography, and when necessary, invasive CSF hypocretin measurement, thus requiring the considerable expenditure of resources^6^. Even so, the diagnosis is often delayed for years, compounding the individual and societal burdens due to the disease^7,74^. Thus, we further asked if our fMRI variability results could differentiate the NT1 cases from HC. Our ROC analysis of the mean variances from AAN and its component nuclei (Fig. 4a, b) indicated that mean Var_frac_ in MREG_vlf_ from AAN (AUC = 0.785) and MRF (AUC = 0.829) were best able to differentiate the NT1 from HC, thus showing promise as a non-invasive bi-classifier. Moreover, we found decreased fMRI signal variability of cardiac- and respiratory-related brain pulsations in the NT1 patients compared to the HC group, as distinct from the increased cardiorespiratory-related pulsations reported in Alzheimer’s disease^32^ and the increased respiratory-related pulsation in epilepsy^31^. Together, these findings suggest that narcolepsy type 1 may have a signature pulsation spectrum that is different from that of other diseases.

BOLD signal variability increases in dementia has a relation to cognitive performance and changes as a function of healthy ageing^32,34,36^. We used Pearson correlation analysis to test for an association between narcolepsy severity scores and our variance results for brain pulsation in the AAN within the NT1 group. This analysis revealed a significant negative correlation between mean-variance in MREG_card_ and individual NSS scores (Fig. 4c), establishing a clinically quantifiable and relevant relation between disease severity (NSS) and our cardiac-related findings. Hypertension reduces arterial pulsatility, thus disrupting perivascular pumping and slowing the transport of CSF in the brain arterial perivascular spaces, which in turn decreases glymphatic flow^26^. Although patients with narcolepsy type 1 are at risk for hypertension and non-dipping nocturnal blood pressure profile^29,75,76^, we found no difference in blood pressure between the NT1 and HC groups in our study. Thus, our results imply that patients with narcolepsy type 1 may have altered brain pulsations that are not explicable by blood pressure differences. Nonetheless, we postulate that effective treatments for hypertension, daytime arousal deficiency, and fragmented nocturnal sleep architecture should prove important to stabilize brain pulsations in patients with narcolepsy type 1. It is tempting to speculate that the present results may be linked to altered glymphatic function in narcolepsy type 1 through suggested changes in the brain CSF dynamics, although this reasoning is indirect in nature.

Our NT1 group had heterogeneous drug treatment regimens that may have confounded our brain pulsation findings. This limitation is difficult to avoid, given the rarity of the disease (prevalence of 1/2000)^9^, and the heavy burden of symptoms in untreated patients, such that we declined to call for a cessation of treatment prior to participation in this study. Furthermore, as the study sample included some non-medicated patients, and others medicated by one or more of the standard psychostimulant drugs, we expect that confounding effects would likely have been obscured by the heterogeneity of medication state. Psychostimulant medications used in the treatment of narcolepsy may affect autonomic activity^77^. However, we found no significant differences between our NT1 and HC groups in indirect measures of autonomic balance (cardiorespiratory rates, systolic/diastolic pressures and mean arterial pressure)^78^, further suggesting that the effect of medication on brain pulsations was likewise modest in the present study. Narcolepsy affects the arousal state, such that untreated patients would be prone to fall asleep in the scanner which in turn may affect brain pulsations. However, all participants were instructed to stay awake with their eyes open in the scanner and their vigilance state was verbally checked before and after the scanning lasting 10 min. Furthermore, most patients were taking arousal-enhancing medication, as noted above. In addition, we recently showed that all physiological brain pulsations (vasomotor, respiratory as well as cardiovascular) increase in non-rapid eye movement sleep in a healthy human brain in contrast to the cardiorespiratory changes in the NT1 data of this study^79^, which further indicates that the pulsation differences in our NT1 group are rather pathologically than vigilance driven. These considerations make it unlikely that the overall vigilance state would explicitly confound our present findings. Future studies of this type might benefit from objective indicators of vigilance, such as an eye camera. The relationship between fMRI signal variability and brain clearance is not without ambiguity. However, there is strong evidence that the high-frequency fMRI signal contains information about the forces driving CSF flux and linked metabolite clearance^19,24,39,56^, thus establishing a connection between the two phenomena. More quantitative investigations are needed to establish better this relationship in humans.

Conventional fMRI is confounded by higher frequency physiological signal sources since events in higher frequency bands become aliased to lower frequencies. Moreover, sampling of the cardiac frequency remains inaccurate in low temporal resolution fMRI, as dictated by the Nyquist theorem^39^. We have shown earlier that 1 Hz cardiac power is mainly detectable with an image repetition time (TR) <0.3 s. Furthermore, the cardiac power is aliased over respiratory power in the central brain bordering CSF. We thus argued that critical analysis of respiratory power changes requires sampling with TR <0.5 s^39^. However, given adequate temporal resolution, conventional BOLD sequences may yield comparable results. Furthermore, slower sequences can detect overall (full band) signal variability changes in healthy aging^34^ and in pathology, i.e., in Alzheimer’s disease^32^, although faster sampling rates are required to characterize accurately the source of the altered signal variability (e.g., cardiorespiratory frequency-related pulsation). To gain sufficient temporal accuracy, we used the ultrafast fMRI sequence MREG, which provides temporal sampling at 10 Hz, well in excess of the human cardiac rate. This enables accurate analysis of individual pulsation frequencies without interference from respiratory-related cardiac heterodynes or harmonics from lower frequencies^80^. Additionally, our high temporal resolution scanning during ten minutes produced 5922 images per study subject, which translates to high statistical power^81^ sufficient for estimating signal variability. Heart rate, heart rate variability, and respiration rate/volume have been shown to elicit BOLD signal changes that affect apparent functional connectivity fluctuations^82–84^. Thus, differences in heart rate and respiration between study groups may introduce confounders to the MREG data. To control for this, we used photoplethysmogram, end-tidal CO2, and MREG data to confirm the absence of significant differences in heart and respiration rates between our NT1 and HC groups (Supplementary Data 1).

With our present results, we cannot directly infer whether less severe narcolepsy type 1 or narcolepsy type 2 can be bi-classified by the ROC from AAN brain pulsations. However, our results lay the groundwork for future investigations and indicate a negative relation between the NSS and cardiac-related brain pulsations in our NT1 group, in whom NSS scores range from 8 to 45 (from milder to more severe), predicting that less severe forms of the disease may be correctly bi-classified by the ROC analysis.

Our method is non-invasive and lasts only ten minutes, which favors its clinical use in centers with fast fMRI capabilities, especially as a potential tool for resolving uncertain diagnoses or avoiding the need for invasive CSF collection for hypocretin assays. We confined the present study to narcolepsy type 1 patients, so it should prove important to apply our methods to narcolepsy type 2 patients (i.e., narcolepsy without cataplexy with largely unknown pathophysiology^9^), as well as other rare sleep-related disorders. Ultrafast fMRI variance analysis might reveal physiological distinctions between the two forms of narcolepsy, or enable differential diagnosis within the disease and discrimination between narcolepsy and other sleep-related symptoms such as idiopathic hypersomnia. Furthermore, it would be interesting to image narcolepsy patients with and without hypertension to examine whether there exists a cumulative effect on brain pulsations.

## Conclusions

We present novel evidence of altered brain pulsations in narcolepsy type 1, as depicted by increased very low frequency and decreased cardiorespiratory-related pulsations. In further analyses, we found a correlation between cardiac-related brain pulsation and disease severity, and that variability in the very low-frequency band shows promise as a diagnostic bi-classifier with clinical relevance. We postulate that these results predict impaired CSF flow that in turn may be linked to altered brain clearance in narcolepsy type 1.

## Supplementary information

Supplementary information

Description of Additional Supplementary Files

Supplementary Data 1

Reporting Summary

## Data Availability

The source data is available in Supplementary Data 1 or upon reasonable request from the corresponding author.

## Code availability

All computations were conducted with preexisting FSL, AFNI, MATLAB, and RStudio functions as described in the Materials and methods section. Code available upon reasonable request.
