# Supplementary material for: Increased very low frequency pulsations and decreased cardiorespiratory pulsations suggest altered brain clearance in narcolepsy
Source: Commun Med (Lond). 2022 Sep 30;2:122. doi: 10.1038/s43856-022-00187-4 (PMC9525269; doi:10.1038/s43856-022-00187-4)
Supplement: Supplementary file 1 — Supplementary information [file 43856_2022_187_MOESM1_ESM.pdf]

Increased very low frequency pulsations and decreased cardiorespiratory pulsations suggest altered brain clearance in narcolepsy

Matti Järvelä[1,2], Janne Kananen[1,2], Vesa Korhonen[1,2], Niko Huotari[1,2], Hanna Ansakorpi[3,4], Vesa Kiviniemi[1,2*]

* Corresponding author:

Vesa Kiviniemi, Prof, MD

vesa.kiviniemi@oulu.fi

1 Department of Diagnostic Radiology, Medical Research Center (MRC), Oulu University Hospital, Oulu, Finland

2 Research unit of Medical Imaging, Physics and Technology, the Faculty of Medicine, University of Oulu, Oulu, Finland

3 Research Unit of Neuroscience, Neurology, University of Oulu, Oulu, Finland

4 Department of Neurology, Oulu University Hospital, Oulu, Finland

Supplementary information

Supplementary Figure 1. Cardiorespiratory frequency comparisons between modalities.

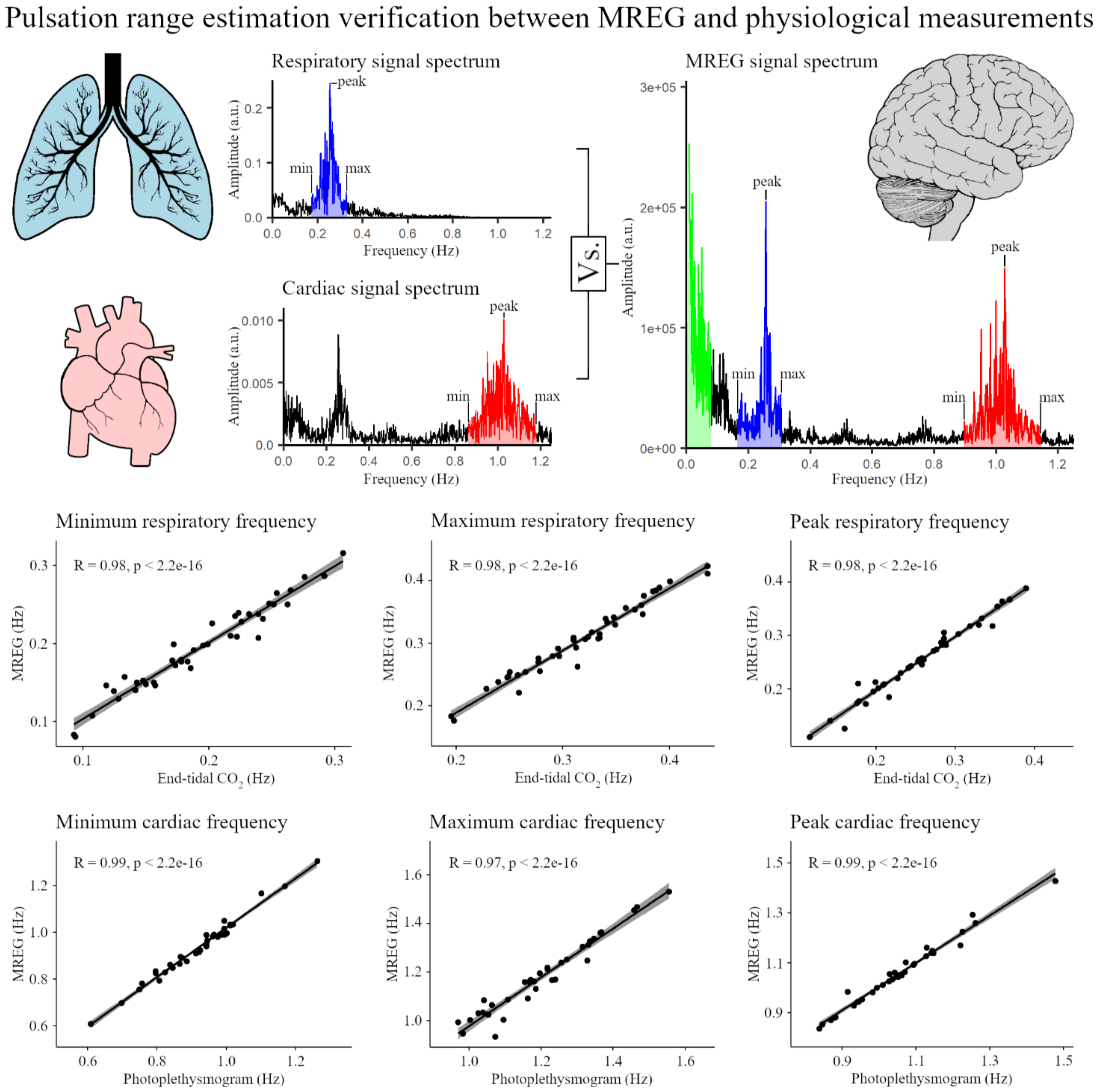

Individual minimum, maximum, and peak values of respiration (blue signal) and cardiac (red signal) pulse derived from end-tidal CO_2 (n = 41 vs. 41) and photoplethysmogram (n = 35 vs. 35), respectively, correlate extremely well with corresponding values estimated from magnetic resonance encephalography (MREG) data. Min = minimum frequency, max = maximum frequency, peak = peak frequency, green signal indicates very low frequency band, black dots denote individual data points, grey are indicates 95% confidence interval, R = Pearson correlation coefficient, p = p-value.

Supplementary Table 1. Brainstem AAN nuclei-wise p-values and statistical information for mean variance.

	$MREG_{vlf}$	$MREG_{resp}$	$MREG_{card}$
DR	p = 0.37; NT1: M = 3377 [Q1: 2371, Q3: 13515]; HC: M = 4367 [Q1: 1238, Q3: 5810]; W = 281; 95% CI [-1636, 3531]	p = 0.35; NT1: M = 5842 [Q1: 2726, Q3: 9489]; HC: M = 7489 [Q1: 4222, Q3: 11564]; W = 201; 95% CI [-4773, 1551]	p = 0.027; NT1: M = 7778 [Q1: 6512, Q3: 13269]; HC: M = 12718 [Q1: 7986, Q3: 30572]; W = 148; 95% CI [-16042, -397]
LC	p = 0.17; NT1: M = 3368 [Q1: 2098, Q3: 6652]; HC: M = 2920 [Q1: 998, Q3: 4103]; W = 301, 95% CI [-551, 2592]	p = 0.68; NT1: M = 5073 [Q1: 2363, Q3: 11717]; HC: M = 5489 [Q1: 3714, Q3: 12880]; W = 224; 95% CI [-3796, 2457]	p = 0.35; NT1: M = 8903 [Q1: 4377, Q3: 11259]; HC: M = 9103 [Q1: 6551, Q3: 21534]; W = 201; 95% CI [-6186, 1907]
MR	p = 0.30; NT1: M = 3755 [Q1: 1917, Q3: 8222]; HC: M = 3091 [Q1: 1288, Q3: 5897]; W = 287; 95% CI [-1027, 2849]	p = 0.99; NT1: M = 4340 [Q1: 2407, Q3: 15805]; HC: M = 6313 [Q1: 2290, Q3: 11598]; W = 241; 95% CI [-3603, 3433]	p = 0.080; NT1: M = 4637 [Q1: 3514, Q3: 7400]; HC: M = 8392 [Q1: 3962, Q3: 17034]; W = 167; 95% CI [-9041, 254]
MRF	p = 0.29; NT1: M = 3648 [Q1: 2366, Q3: 12578]; HC: M = 3086 [Q1: 1428, Q3: 7252]; W = 288; 95% CI [-949, 3835]	p = 0.22; NT1: M = 3481 [Q1: 2056, Q3: 9367]; HC: M = 5911 [Q1: 3576, Q3: 10919]; W = 189; 95% CI [-4068, 944]	p = 0.0031*; r = 0.44; NT1: M = 6563 [Q1: 3348, Q3: 10376]; HC: M =12823 [Q1: 7830, Q3: 26854]; W = 118; 95% CI [-12950, -2017]
PAG	p = 0.18; NT1: M = 6080 [Q1: 3251, Q3: 13544]; HC: M = 4319 [Q1: 1679, Q3: 9889]; W = 300; 95% CI [-1204, 5901]	p = 0.58; NT1: M = 5805 [Q1: 2870, Q3: 10299]; HC: M = 7585 [Q1: 4455, Q3: 12851]; W = 218; 95% CI [-4825, 2408]	p = 0.062; NT1: M = 16018 [Q1: 7867, Q3: 23583]; HC: M = 22116 [Q1: 13035, Q3: 53286]; W = 162; 95% CI [-25053, 551]
PBC	p = 0.13; NT1: M = 3323 [Q1: 2232, Q3: 6146]; HC: M = 2514 [Q1: 1044, Q3: 3950]; W = 307; 95% CI [-344, 2555]	p = 0.86; NT1: M = 4159 [Q1: 2800, Q3: 8087]; HC: M = 4348 [Q1: 2293, Q3: 10477]; W = 250; 95% CI [-2503, 2537]	p = 0.17; NT1: M = 4332 [Q1: 2941, Q3: 5959]; HC: M = 5551 [Q1: 3614, Q3: 12125]; W = 183; 95% CI [-4117, 511]
PO	p = 0.26; NT1: M = 3708 [Q1: 2124, Q3: 7265]; HC: M = 3052 [Q1: 1275, Q3: 5895]; W = 291; 95% CI [-794, 2856]	p = 0.95; NT1: M = 4423 [Q1: 2348, Q3: 14195]; HC: M = 5640 [Q1: 2213, Q3: 10654]; W = 245; 95% CI [-3551, 3462]	p = 0.076; NT1: M = 4177 [Q1: 2852, Q3: 7793]; HC: M = 7695 [Q1: 4136, Q3: 16301]; W = 166; 95% CI [-8850, 253]
PPN	p = 0.48; NT1: M = 2533 [Q1: 1915, Q3: 10433]; HC: M = 2729 [Q1: 973, Q3: 4756]; W = 273; 95% CI [-912, 2002]	p = 0.84; NT1: M = 4143 [Q1: 1759, Q3: 9989]; HC: M = 4723 [Q1: 2216, Q3: 9631]; W = 233; 95% CI [-2602, 2874]	p = 0.039; NT1: M = 2688 [Q1: 1766, Q3: 4022]; HC: M = 3947 [Q1: 2365, Q3: 5928]; W = 154; 95% CI [-2824, -88]
VTA	p = 0.48; NT1: M = 2013 [Q1: 1155, Q3: 6201]; HC: M = 2099 [Q1: 786, Q3: 4041]; W = 273; 95% CI [-981, 1715]	p = 0.81; NT1: M = 5190 [Q1: 1970, Q3: 12736]; HC: M = 3550 [Q1: 2281, Q3: 8281]; W = 253; 95% CI [-1884, 4100]	p = 0.031; NT1: M = 2531 [Q1: 1848, Q3: 4806]; HC: M = 4453 [Q1: 3384, Q3: 7527]; W = 150; 95% CI [-3161, -149]

DR = dorsal raphe, LC = locus caeruleus, MR = median raphe, MRF = midbrain reticular formation, PAG = periaqueductal gray, PBC = parabrachial complex, PO = pontis oralis, PPN = pedunculopontine nucleus, VTA = ventral tegmental area, * = corrected significant p-value ($p < 0.016$), n = 22 vs. 22, M = median, Q1 = 1st quartile, Q3 = 3rd quartile, 95% CI = 95% confidence interval, W = Wilcoxon test statistic, r = Wilcoxon effect size, $MREG_{vlf}$ = very low frequency filtered magnetic resonance encephalography data, $MREG_{resp}$ = respiratory frequency filtered MREG data, $MREG_{card}$ = cardiac frequency filtered MREG data. All p-values displayed with means were calculated with Student's t-test and all p-values displayed with medians were calculated with Wilcoxon rank sum test.

Supplementary Table 2. Brainstem AAN nuclei-wise p-values and statistical information for mean fractional variance.

	$MREG_{vlf}$	$MREG_{resp}$	$MREG_{card}$
DR	p = 0.0050*; r = 0.42; NT1: M = 0.11 [Q1: 0.062, Q3: 0.19]; HC: M = 0.057 [Q1: 0.043, Q3: 0.092]; W = 360, 95% CI [0.013, 0.095]	p = 0.60; NT1: M = 0.11 [Q1: 0.074, Q3: 0.15]; HC: M = 0.098 [Q1: 0.056, Q3: 0.16]; W = 265; 95% CI [-0.031, 0.050]	p = 0.017*; d = 0.75; NT1: µ = 0.18 ± 0.084 SD; HC: µ = 0.24 ± 0.090 SD; 42 df; T = -2.4859; 95% CI [-0.12, -0.012]
LC	p = 0.0017**; r = 0.46; NT1: M = 0.091 [Q1: 0.069, Q3 0.13]; HC: M = 0.055 [Q1: 0.030, Q3: 0.076], W = 373; 95% CI [0.013, 0.069]	p = 0.84; NT1: M = 0.1074 [Q1: 0.075, Q3: 0.15]; HC: M = 0.12 [Q1: 0.057, Q3: 0.17], W = 251; 95% CI [-0.048, 0.040]	p = 0.24; NT1: µ = 0.18 ± 0.082 SD; HC: µ = 0.21 ± 0.082 SD; 42 df; T = -1.1923; 95% CI [-0.079, 0.020]
MR	p = 0.0050**; r = 0.42; NT1: M = 0.12 [Q1: 0.088, Q3: 0.19]; HC: M = 0.062 [Q1: 0.043, Q3: 0.098], W = 360; 95% CI [0.017, 0.093]	p = 0.58; NT1: M = 0.14 [Q1: 0.11, Q3: 0.19]; HC: M = 0.13 [Q1: 0.066, Q3: 0.22], W = 266; 95% CI [-0.046, 0.069]	p = 0.12; NT1: µ = 0.16 ± 0.088 SD; HC: µ = 0.21 ± 0.12 SD; 42 df; T = -1.5685; 95% CI [-0.11, 0.014]
MRF	p = 0.00017*; d = 1.3; NT1: µ = 0.17 ± 0.089 SD; HC: µ = 0.083 ± 0.037 SD; 27.836 df; T = 4.3398; 95% CI [0.047, 0.13]	p = 0.77; NT1: M = 0.13 [Q1: 0.090, Q3: 0.17]; HC: M = 0.11 [Q1: 0.077, Q3: 0.17], W = 255; 95% CI [-0.041, 0.044]	p = 0.012*; d = 0.79; NT1: µ = 0.19 ± 0.10 SD; HC: µ = 0.27 ± 0.099 SD; 42 df; T = -2.6168; 95% CI [-0.14, -0.018]
PAG	p = 0.0036*; r = 0.43; NT1: M = 0.12 [Q1: 0.063, Q3: 0.24]; HC: M = 0.062 [Q1: 0.033, Q3: 0.090], W = 364; 95% CI [0.015, 0.11]	p = 0.27; NT1: M = 0.089 [Q1: 0.066, Q3: 0.11]; HC: M = 0.062 [Q1: 0.042, Q3: 0.12], W = 290; 95% CI [-0.016, 0.047]	p = 0.031*; d = 0.67; NT1: µ = 0.20 ± 0.096 SD; HC: µ = 0.27 ± 0.11 SD; 42 df; T = -2.2344; 95% CI [-0.13, -0.0068]
PBC	p = 0.0033**; r = 0.44; NT1: M = 0.12 [Q1: 0.095, Q3: 0.18]; HC: M = 0.077 [Q1: 0.044, Q3: 0.10], W = 365; 95% CI [0.015, 0.087]	p = 0.88; NT1: M = 0.14 [Q1: 0.10, Q3: 0.16]; HC: M = 0.13 [Q1: 0.075, Q3: 0.22], W = 249; 95% CI [-0.048, 0.045]	p = 0.092; NT1: µ = 0.15 ± 0.077 SD; HC: µ = 0.19 ± 0.082 SD; 42 df; T = -1.7222; 95% CI [-0.090, 0.0071]
PO	p = 0.0068**; r = 0.40; NT1: M = 0.12 [Q1: 0.086, Q3: 0.19]; HC: M = 0.065 [Q1: 0.041, Q3: 0.10], W = 356; 95% CI [0.015, 0.093]	p = 0.67; NT1: M = 0.13 [Q1: 0.11, Q3: 0.18]; HC: M = 0.13 [Q1: 0.073, Q3: 0.22], W = 261; 95% CI [-0.044, 0.064]	p = 0.11; NT1: µ = 0.16 ± 0.088 SD; HC: µ = 0.21 ± 0.12 SD; 42 df; T = -1.6196; 95% CI [-0.12, 0.013]
PPN	p = 0.0054**; r = 0.41; NT1: M = 0.15 [Q1: 0.12, Q3: 0.26]; HC: M = 0.11 [Q1: 0.065, Q3: 0.14], W = 359; 95% CI [0.016, 0.11]	p = 0.99; NT1: µ = 0.20 ± 0.10 SD; HC: µ = 0.20 ± 0.10 SD; 42 df; T = 0.0026758; 95% CI [-0.062, 0.063]	p = 0.041; NT1: M = 0.12 [Q1: 0.080, Q3: 0.22]; HC: M = 0.17 [Q1: 0.14, Q3: 0.21], W = 155; 95% CI [-0.092, -0.0059]
VTA	p = 0.098; NT1: M = 0.12 [Q1: 0.080, Q3: 0.23]; HC: M = 0.010 [Q1: 0.068, Q3: 0.13], W = 313; 95% CI [-0.0052, 0.083]	p = 0.79; NT1: M = 0.18 [Q1: 0.14, Q3: 0.28]; HC: M = 0.20 [Q1: 0.15, Q3: 0.28], W = 254; 95% CI [-0.055, 0.070]	p = 0.033; NT1: M = 0.11 [Q1: 0.069, Q3: 0.26]; HC: M = 0.20 [Q1: 0.17, Q3: 0.34], W = 151; 95% CI [-0.13, -0.011]

DR = dorsal raphe, LC = locus caeruleus, MR = median raphe, MRF = midbrain reticular formation, PAG = periaqueductal gray, PBC = parabrachial complex, PO = pontis oralis, PPN = pedunculopontine nucleus, VTA = ventral tegmental area, * = corrected significant p-value (p < 0.033), ** = corrected significant p-value (p < 0.016), n = 22 vs. 22, µ = mean, M = median, Q1 = 1st quartile, Q3 = 3rd quartile, 95% CI = 95% confidence interval, W = Wilcoxon test statistic, r = Wilcoxon effect size, T = t-test statistic, d = Cohen's d effect size. $MREG_{vlf}$ = very low frequency filtered magnetic resonance encephalography data, $MREG_{resp}$ = respiratory frequency filtered MREG data, $MREG_{card}$ = cardiac frequency filtered MREG data. All p-values displayed with means were calculated with Student's t-test and all p-values displayed with medians were calculated with Wilcoxon rank sum test.
