# Supplementary material for: Increased very low frequency pulsations and decreased cardiorespiratory pulsations suggest altered brain clearance in narcolepsy
Source: Commun Med (Lond). 2022 Sep 30;2:122. doi: 10.1038/s43856-022-00187-4 (PMC9525269; doi:10.1038/s43856-022-00187-4)
Supplement: Supplementary file 2 — Description of Additional Supplementary Files [file 43856_2022_187_MOESM2_ESM.pdf]

Description of Additional Supplementary Files

File Name: Supplementary Data 1

Description: Source data including AAN- and nuclei-wise variances, cardiorespiratory estimations, motion, blood pressure and NSS values
