# Supplementary material for: Increased very low frequency pulsations and decreased cardiorespiratory pulsations suggest altered brain clearance in narcolepsy
Source: Commun Med (Lond). 2022 Sep 30;2:122. doi: 10.1038/s43856-022-00187-4 (PMC9525269; doi:10.1038/s43856-022-00187-4)
Supplement: Supplementary file 4 — Reporting Summary [file 43856_2022_187_MOESM4_ESM.pdf]

nature research

Corresponding author(s): Vesa Kiviniemi, Prof., MD.
Oulu Functional Neuroimaging
vesa.kiviniemi@oulu.fi

Last updated by author(s): Sep 8, 2022

Reporting Summary

Nature Research wishes to improve the reproducibility of the work that we publish. This form provides structure for consistency and transparency in reporting. For further information on Nature Research policies, see our Editorial Policies and the Editorial Policy Checklist.

Statistics

For all statistical analyses, confirm that the following items are present in the figure legend, table legend, main text, or Methods section.

n/a	Confirmed	
☐	☒	The exact sample size (*n*) for each experimental group/condition, given as a discrete number and unit of measurement
☐	☒	A statement on whether measurements were taken from distinct samples or whether the same sample was measured repeatedly
☐	☒	The statistical test(s) used AND whether they are one- or two-sided<br>*Only common tests should be described solely by name; describe more complex techniques in the Methods section.*
☐	☒	A description of all covariates tested
☐	☒	A description of any assumptions or corrections, such as tests of normality and adjustment for multiple comparisons
☐	☒	A full description of the statistical parameters including central tendency (e.g. means) or other basic estimates (e.g. regression coefficient) AND variation (e.g. standard deviation) or associated estimates of uncertainty (e.g. confidence intervals)
☐	☒	For null hypothesis testing, the test statistic (e.g. *F*, *t*, *r*) with confidence intervals, effect sizes, degrees of freedom and *P* value noted<br>*Give P values as exact values whenever suitable.*
☒	☐	For Bayesian analysis, information on the choice of priors and Markov chain Monte Carlo settings
☒	☐	For hierarchical and complex designs, identification of the appropriate level for tests and full reporting of outcomes
☒	☐	Estimates of effect sizes (e.g. Cohen's *d*, Pearson's *r*), indicating how they were calculated

Our web collection on statistics for biologists contains articles on many of the points above.

Software and code

Policy information about availability of computer code

Data collection	Siemens Skyra 3T MRI machine
Data analysis	FSL 5.09, AFNI version 18.0.05, MATLAB version R2019b, RStudio version 1.3.1093

For manuscripts utilizing custom algorithms or software that are central to the research but not yet described in published literature, software must be made available to editors and reviewers. We strongly encourage code deposition in a community repository (e.g. GitHub). See the Nature Research guidelines for submitting code & software for further information.

Data

Policy information about availability of data

All manuscripts must include a data availability statement. This statement should provide the following information, where applicable:
- Accession codes, unique identifiers, or web links for publicly available datasets
- A list of figures that have associated raw data
- A description of any restrictions on data availability

The source data is available in the Supplementary Data 1 or upon reasonable request from the corresponding author.

Field-specific reporting

Please select the one below that is the best fit for your research. If you are not sure, read the appropriate sections before making your selection.

☒ Life sciences ☐ Behavioural & social sciences ☐ Ecological, evolutionary & environmental sciences

For a reference copy of the document with all sections, see nature.com/documents/nr-reporting-summary-flat.pdf

Life sciences study design

All studies must disclose on these points even when the disclosure is negative.

Sample size	Narcolepsy is a rare condition and thus large study populations are hard to accrue. During data collection, all narcolepsy type 1 patients (from Oulu University Hospital's electronical patient registry at that time frame) were called and asked to participate in the study, and all who agreed were then imaged. After exclusion for corrupted data, the sample size for this study settled to 22. The authors deemed sample size over 20 acceptable.
Data exclusions	Data from one patient was excluded as the fMRI data was visibly corrupted due to failure in off-resonance correction.
Replication	We ran the randomise analysis twice with the same settings and observed the same results on both occasions. The raw data is secured on our research groups own server behind password.
Randomization	Randomization of participants is not needed in this study as we compare patients with narcolepsy to healthy controls (individuals themselves are of course unrecognizable) and the study groups are assigned thusly.
Blinding	Blinding of study groups was not relevant in this study. No interventions were assessed.

Reporting for specific materials, systems and methods

We require information from authors about some types of materials, experimental systems and methods used in many studies. Here, indicate whether each material, system or method listed is relevant to your study. If you are not sure if a list item applies to your research, read the appropriate section before selecting a response.

Materials & experimental systems

n/a	Involved in the study	
☒	☐	Antibodies
☒	☐	Eukaryotic cell lines
☒	☐	Palaeontology and archaeology
☒	☐	Animals and other organisms
☐	☒	Human research participants
☒	☐	Clinical data
☒	☐	Dual use research of concern

Methods

n/a	Involved in the study	
☒	☐	ChIP-seq
☒	☐	Flow cytometry
☐	☒	MRI-based neuroimaging

Human research participants

Policy information about studies involving human research participants

Population characteristics	Twenty-two narcolepsy type 1 patients (12 females, mean age 28.1 ± 8.9 SD). Four of the NT1 patients were unmedicated and 18 were medically treated for daytime sleepiness and cataplexy.<br><br>Twenty-two sex- and age-matched (12 females, mean age 28.2 ± 8.9 SD) healthy controls with no continuous medication were used as a control group.
Recruitment	A registry run from the Oulu University Hospital's electronic patient records for patients diagnosed with all-type narcolepsy was conducted. All the diagnoses were reassessed with International Classification of Sleep Disorders 3th edition in order to identify up to date narcolepsy type 1 diagnoses. All patients with confirmed narcolepsy type 1 diagnose were then called/ interviewed and asked to participate in the study resulting in a group of 23 narcolepsy type 1 patients (one excluded in the analysis stage due to corrupted data).<br><br>Age- and sex-matched healthy controls were recruited from general population by advertisement.
Ethics oversight	Ethical Committee of Medical Research in the Northern Ostrobothnia District of Finland

Note that full information on the approval of the study protocol must also be provided in the manuscript.

Magnetic resonance imaging

Experimental design

Design type	Resting-state fMRI
Design specifications	10 min resting-state fMRI resulting in 5922 full brain images
Behavioral performance measures	We used resting-state design without any task.

Acquisition

Imaging type(s)	Functional, structural
Field strength	3T
Sequence & imaging parameters	Pulse sequence: gradient echo, imaging type: spiral, FOV: 192 mm, matrix size: 64 x 64 x 64 x 5922, slice thickness 3 mm, orientation: radiological, TE = 36 , TR = 100 ms, FA = 25.
Area of acquisition	Whole brain

Diffusion MRI ☐ Used ☒ Not used

Preprocessing

Preprocessing software	FSL version 5.09. The data were high-pass filtered with a cut-off frequency of 0.008 Hz (125 s). Motion correction was carried out using FSL MCFLIRT. Brain extraction for 3D MPRAGE volumes was performed with FSL Brain Extraction TOOL (BET) using neck and bias-field correction and the following parameters: fractional intensity = 0.20-0.22 and threshold gradient = 0.05-0.25. Images were spatially smoothed with 5 mm full width and half maximum Gaussian kernel using fslmaths. MREG images were aligned to 3D anatomical images (full-search, 12 degree of freedom (DOF)) and to Montreal Neurological Institute (MNI 152) 4mm3 standard space (full-search, 12 DOF) as a preprocessing step in FSL Multivariate Exploratory Linear Optimized Decomposition into Independent Components tool.
Normalization	MREG images were aligned to 3D anatomical images (full-search, 12 degree of freedom (DOF)) and to Montreal Neurological Institute (MNI 152) 4mm3 standard space (full-search, 12 DOF) as a preprocessing step in FSL Multivariate Exploratory Linear Optimized Decomposition into Independent Components tool. In the analysis stage, variance results were registered to 3 mm MNI152 standard space and with nucleus-wise comparisons to 1 mm MNI152 standard space to comply.
Normalization template	MNI152 4/3/1 mm3
Noise and artifact removal	MCFLIRT, careful exclusion of statistical difference in movement parameters (mean absolute and mean relative movement as well as mean frame-wise displacement).
Volume censoring	Not used.

Statistical modeling & inference

Model type and settings	Randomise with 10,000 iterations: Conditional Monte Carlo random permutations implementing family-wise error-corrected threshold-free cluster enhancement correction in both directions separately.
Effect(s) tested	No task or stimulus used in this study.

Specify type of analysis: ☐ Whole brain ☐ ROI-based ☒ Both

Anatomical location(s)	Predefined ascending arousal network region of interest as described in: Edlow, B. L. et al. Neuroanatomic Connectivity of the Human Ascending Arousal System Critical to Consciousness and Its Disorders. J Neuropathol Exp Neurol 71, 531–546 (2012).
Statistic type for inference (See Eklund et al. 2016)	Voxel-wise, Randomise (see above)
Correction	For fMRI data: Conditional Monte Carlo random permutations implementing family-wise error-corrected threshold-free cluster enhancement correction in both directions separately.<br><br>In other instances Benjamini-Hochberg procedure was used to control for multiple comparisons.

Models & analysis

n/a	Involved in the study
☒	☐ Functional and/or effective connectivity
☒	☐ Graph analysis
☒	☐ Multivariate modeling or predictive analysis
